# Selecting accurate post-elimination monitoring tools to prevent reemergence of urogenital schistosomiasis in Morocco: a pilot study

**DOI:** 10.1186/s40249-017-0289-z

**Published:** 2017-04-06

**Authors:** Abdelaali Balahbib, Fatima Amarir, Paul L.A.M. Corstjens, Claudia J. de Dood, Govert J. van Dam, Amina Hajli, Meryem Belhaddad, Bouchra El Mansouri, Abderrahim Sadak, Mohamed Rhajaoui, El Bachir Adlaoui

**Affiliations:** 1 National Reference Laboratory for Schistosomiasis and Malacology, National Institute of Hygiene, Agdal, Rabat Morocco; 2 Faculty of Science, Laboratory of Zoology and General Biology, Agdal, Rabat Morocco; 3 Laboratory of Parasitology and Malacology, Institute of Nursing Professions and Health Techniques, Casablanca, Morocco; 4 Department of Molecular Cell Biology, Leiden University Medical Center, Leiden, The Netherlands; 5 Department of Parasitology, Leiden University Medical Center, Leiden, The Netherlands

**Keywords:** Schistosomiasis, Transmission stop, Elimination, Monitoring, Antibody test, Antigen test, Circulating Anodic Antigen (CAA), Active infection

## Abstract

**Background:**

After alleged stop of transmission of schistosomiasis and further down the line in post elimination settings, sensitive tools are required to monitor infection status to prevent potential re-emergence. In Rahala, where transmission cycle of *Schistosoma haematobium* is interrupted since 2004 but where 30% of snails are still infected by *S. bovis*, potential human *S. bovis* infection can’t be excluded. As methods based on egg-counts do not provide the required sensitivity, antibody or antigen assays are envisaged as the most appropriate tools for this type of monitoring.

**Methods:**

In this pilot study, the performances of three assays were compared: two commercially available antibody tests (ELISA and haemagglutination format) indicating exposure, and an antigen test (lateral flow strip format) demonstrating active infection. All 37 recruited study participants resided in Rahala (Akka, province Tata, Morocco). Participants had been diagnosed and cured from schistosomiasis in the period between 1983 and 2003. In 2015 these asymptomatic participants provided fresh clinical samples (blood and urine) for analysis with the aforementioned diagnostics tests.

**Results:**

No eggs were identified in the urine of the 37 participants. The haemagglutination test indicated 6 antibody positives whereas the ELISA indicated 28 antibody positives, one indecisive and one false positive. ELISA and haemagglutination results matched for 18 individuals, amongst which 5 out of 6 haemagglutination positives. With the antigen test (performed on paired serum and urine samples), serum from two participants (cured 21 and 32 years ago) indicated the presence of low levels of the highly specific *Schistosoma* circulating anodic antigen (CAA), demonstrating low worm level infections (less than 5 pg/ml corresponding to probably single worm pair). One tested also CAA positive with urine. ELISA indicated the presence of human anti-*Schistosoma* antibodies in these two CAA positive cases, haemagglutination results were negative.

**Conclusions:**

To prevent reemergence of schistosomiasis in Morocco current monitoring programs require specific protocols that include testing of antibody positives for active infection by the UCP-LF CAA test, the appropriate diagnostic tool to identify *Schistosoma* low grade infections in travelers, immigrants and assumed cured cases. The test is genus specific will also identify infections related to *S. bovis*.

**Electronic supplementary material:**

The online version of this article (doi:10.1186/s40249-017-0289-z) contains supplementary material, which is available to authorized users.

## Multilingual abstracts

Please see Additional file 1 for translations of the six official working languages of the United Nations.

## Background

*Schistosoma haematobium* is responsible for a heavy burden of disease affecting more than 100 million people in sub-Saharan Africa [1, 2]. Effective transmission control of the infection includes accurate (high sensitivity) diagnosis, (preventive) chemotherapy, snail control, sanitation, safe water supplies, and human behavioral change strategies [3]. Morocco, after nearly three decades of effort, was successful in the elimination of urogenital schistosomiasis caused by *S. haematobium*. Since 2004 no new local cases were reported [4]. In 2009 validation of interruption of transmission was commenced with the initiation of a national serological survey (using enzyme-linked immunoelectrotransfer blot, EITB) screening for human antibodies against *S. haematobium* in children, followed by a national molecular malacology survey analyzing the prevalence of infected snails (the intermediate host). The results confirmed interruption of transmission and indicated progress towards elimination as it showed that none of children or the collected snails was infected by *S. haematobium* [5, 6]. However, given that the exact parasite life spans and the distribution of the post-treatment antibody responses across the whole population are not fully understood [1, 7], prevention of reemerging required a vigilant survey strategy. It seems prudent to carefully monitor travelers and immigrants from endemic countries and other potentially high risk groups.

Various protocols for the diagnosis and surveillance of urogenital schistosomiasis have been proposed but none with optimal performance characteristics for sensitive and specific point-of-care (POC) applications [8]. Rapid *Schistosoma* anti-egg antibody strip assays for POC applications have been described [9] and may even be used with non-invasive bodily fluids as urine and saliva. Moreover, diagnosis by detecting specific antibodies seems to be more sensitive than the traditional method detection of eggs in urine [10]. In post-transmission and elimination area, antibody detection demonstrating exposure (not active infections) to the pathogen might be suitable for the group born after transmission stop. For older and previously infected individuals [11–13], antibody detection methods will not be useful as one needs to distinguish past cured infections from current ongoing active infections.

In order to incorporate antibody diagnosis in routine clinical laboratory practice, a robust easy to use, medium to high throughput, sensitive and specific test is needed. Unfortunately, the previously successfully evaluated enzyme-linked immunoelectrotransfer blot (EITB) is not readily available for large scale testing because of the high cost of the specific microsomal antigens used for antibody-capture. Only a few other serological antibody tests for schistosomiasis are commercially available but none of them have been evaluated for use in post elimination settings. More recent molecular diagnostics that target schistosome egg DNA isolated from urine offering high sensitivity and specificity are available, but these methods are still costly, do rely on the presence of eggs, and require significant laboratory infrastructure including qualified staff [8]. A better alternative is the diagnostic test to determine active infections with any *Schistosoma* species (including the veterinarian species) by detection of a schistosome-derived (regurgitated) genus-specific carbohydrate antigen. This lateral flow (LF) based test applies a novel ultrasensitive fluorescent label (upconverting phosphor, UCP) for detection of the *Schistosoma* circulating anodic antigen (CAA) in human circulation and can be used with various body fluids. It allows convenient storage and worldwide shipping at ambient temperature in its current user-friendly dry reagent format [14]. Enhanced sensitivity of this UCP-LF CAA strip testis achieved utilizing centrifugal filtration devices which permit larger sample input. The analysis of a sample volume of 0.5 ml serum or 2 ml urine is believed to allow detection of single worm infections [15]; these two assays are referred to respectively as the SCAA500 and UCAA2000 test, with ‘S’ indicting serum, ‘U’ urine and the number the amount of sample (in μl) concentrated and analyzed on the strip. The amount of sample input is flexible, but relates directly to the achieved analytical sensitivity.

In the current study, three assays were assessed for their potential to accurately evaluate the present status of past schistosomiasis cases: i) indirect haemagglutination assay (IHA), an antibody test; ii) ELISA antibody test; iii) lateral flow antigen test. Both the IHA and ELISA antibody test are based on detection of the presence of human antibody against adult worm antigens, detecting past exposure and ongoing infections. The UCP-LF CAA tests detects *Schistosoma* worm antigen (CAA), and as CAA is rapidly cleared is specific for active infections; testing was performed with paired samples, 150 μl serum and 475 μl urine (SCAA150 and UCAA475) allowing detection of low grade infections. Primary goal of this study is to evaluate diagnostic tools for an appropriate and affordable strategy and protocol for accurate monitoring of post-elimination settings, specifically to prevent reemerging of urogenital schistosomiasis in Morocco. Individuals enrolled in this study allowed assessment of the diagnostic tests for monitoring past urinary schistosomiasis cases considered cured before transmission-stop was declared in 2004.

## Methods

### Study area

The pilot study was conducted in Tata province, located in south-west Morocco. This province has been one of the oldest and largest foci of urogenital schistosomiasis in Morocco. In 1983, a total of 3 371 cases were detected there, with an incidence rate of 34.4 cases per 1 000 residents. In Tata, we selected Akka (more specific, Rahala area) for sampling, in this area the last known cases of schistosomiasis were detected [16]. In 2003, schistosomiasis in Akka accounted for 60% (75 cases) of the total number of cases reported in Morocco. Interruption of transmission was claimed in 2004 and since then no new active autochthonous schistosomiasis cases have been reported. In 2015, serological and molecular-malacology survey showed that none of the children and the collected snails were infected by *S. haematobium,* however about 30% of snails were infected by *S. bovis* [6].

### Patients and samples

In March 2015, a list of 100 past patients for urinary schistosomiasis treated at Rahala and considered cured, was compiled and archived clinical data collected. Inclusion criteria was: being listed in the register of cases infected and treated and always living in Tata; for negative control samples individuals stated that they had never had water contact in endemic areas.

The applied inclusion criteria were: being listed in the register of cases infected and treated, and have always lived in Tata since the drug treatment. Individuals not living in Tata were excluded. Negative controls stated that they had never had water contact in endemic areas.

An administrator of parasitic diseases contacted the 100 individuals to inform them regarding the objectives of the current parasitological survey including a new high sensitivity diagnostic screening tool.

The administrator first contacted all cases detected in 2003, and then continued with preceding years until 100individuals agreed to participate to the survey. Unfortunately, at the survey week, only 37 individuals of the 100 concurred individuals participated. From these individuals, age, sex and location were recorded and 5 ml whole blood for serum and 50 ml urine was collected. Urine was used for *Schistosoma* egg determination, blood was tested for detection of human antibodies against *Schistosoma* as well as detection of *Schistosoma* antigen, CAA (circulating anodic antigen). Eggs and CAA are indicative for active (ongoing) *Schistosoma* infection, whereas antibody detection is indicative for exposure but cannot distinguish between past (cured) and current infection. Negative control samples were included obtained from five individuals (sample id T1–T5) from a non-endemic region (Rabat); individuals stated that they had never had water contact in endemic areas. A high positive control included (sample id T6) was obtained from CDC and was used in the last national serological survey in 2009 [5].

### Parasitological examination, egg detection

All individuals were invited to a communal health center, where the parasitological examination of urine (detection of eggs) was conducted by a team of trained laboratory technicians. From each study participant, 50 ml urine was obtained between 10:00 AM and 2:00 PM, after physical exercise, and collected in properly labeled disposable containers. Containers were centrifuged for 2 mins at 2 000 rpm, and the pellet was thereafter examined microscopically for the presence of the characteristic *S. haematobium* egg using 10× and 40× objectives. Urine samples containing schistosome egg(s) were recorded as positive; absence of eggs of schistosomes was considered negative [17]. Samples (40 ml) were packed carefully and transported to the laboratory in a cooler at 4 °C for storage at −20 °C. Aliquots of 450 μl were shipped to the Netherlands on dry ice for UCP-LF CAA testing at Leiden University Medical Center (LUMC).

### Serological assays, antibody detection by IHA and ELISA

Blood samples were centrifuged for 10 min at 1 500 rpm to collect serum. From each individual, 2 ml of serum was aliquoted over two microfuge tubes. Samples were properly packed, cooled and transferred to the laboratory of the National Institute of Hygiene at Rabat for antibody analysis. The presence of human antibodies against *S. haematobium* was in vestigated with two assays: i) schistosomiasis Fumouze, IHA test from Fumouze Diagnostics (Levallois-Perret, France); ii) the *Schistosoma* IgG-ELISA detecting antibodies reactive with soluble antigen from male and female worms of the Puerto Rico *Schistosoma* strain from NovaTec Immundiagnostica (Dietzenbach, Germany). As reported by Kinke et al., the combination of IHA and ELISA is informative for diagnosis of imported schistosomiasis in non-endemic areas [18].

The IHA test kit was used according to the manufacturer’s instruction. Briefly: 50 μl of a 1:20 initial dilution of each serum was subjected to further two-fold serial dilutions, and 10 μl of sheep red blood cells sensitized with *S. mansoni* adult WA was added to each diluted sample. Positive and negative control sera and non-sensitized red blood cells were included in each test as controls for naturally occurring antibodies [10]. All sera were tested in duplicate. The results were evaluated with a cutoff of 1:80.

The ELISA antibody test provides a qualitative result and detects IgG class antibodies against *Schistosoma* in humans and requires a sample input of 10 μl serum. The immune complex formed by the bound conjugate is visualized by adding Tetramethylbenzidine (TMB) substrate. Absorbance was measured at 450/620 nm within 30 min after addition of the stop solution. Samples were classified as positive, negative, or indeterminate according to the manufacturer’s cutoff values.

### *Schistosoma* antigen detection in serum and urine by UCP-LF CAA

Serum and urine samples were sent to LUMC (the Netherlands) for examination with the UCP-LF CAA test. Serum samples included six control samples labeled T1-6, with T1-5 being negative controls and T6 being a high positive control. Testing at LUMC was done single blind, without specific sample information. Serum samples were tested with the SCAA150 assay and urine samples were tested with the UCAA475 assay [9]. Briefly, 150 μl of serum (SCAA150) or 475 μl of urine (UCAA475) was extracted with an equal volume of 4% (w/v) TCA. Samples were centrifuged and the clear supernatant (200 and 950 μl for serum and urine, respectively) concentrated to 20 μl using 0.5 mL Amicon Ultra Centrifugal Filter Devices (10 kDa cutoff Ultra-0.5 columns, Millipore Corp); urine samples required an extra loading step as the TCA supernatant exceeded the maximum loading capacity of the 0.5 ml columns. Concentrated samples (20 μl) were tested in singlet with the UCP-LF CAA strip assay as described previously [9] with a lower limit of detection of 2.5 and 0.5 pg/ml CAA in serum and urine, respectively. Standard series of CAA spiked in normal human serum or urine were processed along with the clinical samples allowing accurate determination of CAA concentrations.

## Results

### Study group

Fresh clinical samples from former urinary schistosomiasis cases (identified, treated and considered cured in the period 1983 through 2003) were analyzed for the presence eggs, anti-*Schistosoma* antibody and *Schistosoma* antigen. All participants resided in Akka (Rahala area) and were considered healthy in 2015. Of the 37 participants, 65% (*n* = 24) were male and 35% (*n* = 13) were female. The age distribution when sampling for the current study was: 59% 21–40 years of age, 33% 41–60 years and 8% over 61 years. In the past, when infection was diagnosed and treated (between 1983 and 2003), the age distribution of this group was 8% under 7 years, 59% (7–14), 30% (15–49), 3% above 50 (eldest being 75).

#### *Schistosoma* egg detection in urine

Parasitology, microscopic examination of the sediment of the urine samples, indicated that none of individuals harbored *Schistosoma* eggs.

#### Anti-*Schistosoma* antibody detection in serum

Serology as examined by IHA and ELISA indicated the presence of antibodies against *S. haematobium* in 6 (16%) and 28 (76%) subjects, respectively (Table 2). Note that the number of positives for the IHA test would decrease to 1 (ID# 22), when a cutoff threshold of 1:160 was used as suggested by the manufacturer. The 5 extra IHA positives (ID# 8, 9, 10, 30 and 33) all tested positive with the ELISA, implying that the lower cutoff threshold was acceptable. When combining IHA and ELISA testing 29 individuals were antibody positive.

The positive control sample tested positive with both assays. The 5 egg-negative controls without schistosomiasis background from Rabat (a non-endemic region) were antibody negative except for one sample (ID# T1) which returned an antibody positive test result with the ELISA (Table 3).

#### *Schistosoma* antigen detection in urine and serum

**Fig. 1:**
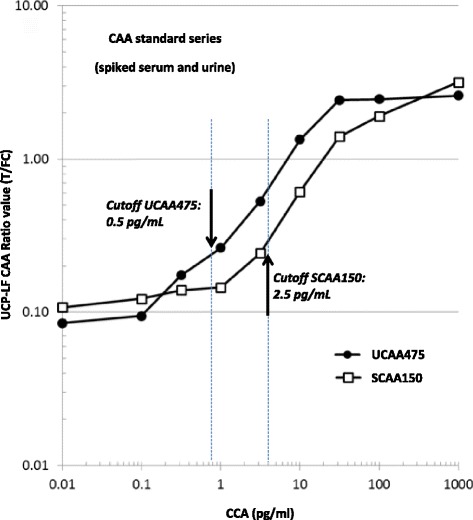
CAA standard series in serum and urine analyzed with UCP-LF. Standard series of CAA spiked in urine and serum. Analyzed with the *Schistosoma* UCP-LF CAA concentration assay utilizing a sample input of 150 μl serum or 475 μl urine, respectively the SCAA150 and UCAA475 test. CAA concentrations are plotted (double logarithmic graph) against the Ratio value obtained with the UCP-LF CAA test. The Ratio value is the signal measured at the Test line (T, CAA capture line) divided by the Flow Control (FC) line on the LF strip

Serum and urine samples were analyzed for the presence of CAA using a sample input of 150 (SCAA150) or 475 (UCAA475) μl, serum and urine respectively. Standard series of CAA spiked in negative human serum and urine were tested alongside and used to calculate CAA levels in the clinical samples (Fig. 1).

#### Serum

Samples from two individuals (ID# 26 and 35) scored CAA levels in the SCAA150 test above the serum cutoff threshold of 2.5 pg/ml; 4.2 and 3.5 pg/mL, respectively. Both samples were also antibody positive with the ELISA test. For three individuals (ID# 13, 15 and 16) the amount of serum was not sufficient to perform the test. All other samples scored below cutoff threshold (Tables 1 and 2). Negative control samples all scored below the cutoff threshold (Table 3) with an average value of 0.84 and highest value of 1.7 (ID# T5). The positive control T6 indicated a serum CAA level >100 pg/ml.

**Table 1:** Participant information and diagnostic test results

Participant information					Diagnostic test results^a^				
ID	Age	Gender	Diagnosed	YAD^b^	Microscopy	IHA	ELISA	CAA Urine	CAA Serum
26	32	Male	1994	21	Negative	Negative	Positive	Positive	Positive
35	47	Male	1983	32	Negative	Negative	Positive	Negative	Positive

^a^Diagnostic tests results from analysis performed in 2015

^b^YAD: number of years after initial diagnosis and drug treatment

**Table 2:** Test results clinical samples

	Diagnostic test		
Result	Positive	Indecisive	Negative
Microscopy	0 (0%)	0 (0%)	37 (100%)
IHA	6 (16.2%)	0 (0%)	31 (83.8%)
ELISA	28 (75.7%)	1 (2.7%)	8 (21.6%)
UCAA474	1 (2.7%)	0 (0%)	35 (97.3)%
SCAA150	2 (5.4%)	0 (0%)	33 (94.6%)

*S. haematobium* past infections (*n* = 37); supposed cured

**Table 3:** Test results negative controls

	Diagnostic test		
Result	Positive	Indecisive	Negative
Microscopy	0 (0%)	0 (0%)	5 (100%)
IHA	0 (0%)	0 (0%)	5 (100%)
ELISA	1 (10%)	0 (0%)	4 (80%)
UCAA475	0 (0%)	0 (0%)	5 (100%)
SCAA150	0 (0%)	0 (0%)	5 (100%)

*S. haematobium* negative controls (*n* = 5); supposed exposure free

#### Urine

Only one urine sample (ID# 26) scored a CAA level in the UCAA475 test above the urine cutoff threshold; 0.85 pg/ml. This CAA-positive urine sample had a matching CAA-positive serum sample and an ELISA antibody positive test result. All other samples scored below the 0.5 pg/ml cutoff threshold for the UCAA950 urine test.

## Discussion

Accurate diagnosis of *Schistosoma* infections challenging in low endemic setting and countries as Morocco where transmission stop is declared and the urogenital schistosomiasis is assumed to be eliminated. Diagnosis of infection is classically based on the detection of parasite eggs in urine or in feces. However, the method clearly isn’t sensitive enough to discover infections with low worm burden [19]. Our study applied two commercially available serological assays (IHA and ELISA) and an ultrasensitive research tool, UCP-LF CAA test detecting the presence of *Schistosoma* antigens in human blood (circulation) and other bodily fluids [9, 20].

In current study group, the classic parasitological methods on urines samples from 37 old schistosomiasis cases (treated and considered cured up to 32 years; confirmed by absence of hematuria) did not reveal *S. haematobium* eggs. It supported, as expected, the absence of active egg shedding by old schistosomiasis cases in this areas. When using antibody serology, a diagnostic detecting exposure (active and past infection [21]), only 8 (21.6%) out of 37 samples tested antibody negative. Consequently, 29 (78.4%) tested antibody positive with either the IHA or ELISA or both. We note however that heterogenic distribution of the post-treatment antibody responses across population may become an issue when using antibody serology as a diagnostic tool to monitor elimination and potential re-emergence.

A statistically relevant comparison of IHA and ELISA in this study has limitations as a consequence of the relatively small sample set and complication to determine a proper cutoff threshold for the IHA test. Initially following the manufacturer’s directions, only 1 antibody positive case was identified IHA, a sample from a case that tested negative with the ELISA. Using an adjusted (lower) cutoff threshold, 5 more positives were identified, all confirmed with ELISA. Obviously, as the IHA indicated threshold may not have been optimal for this sample set, a larger confirmed negative control group would have been required to determine the appropriate thresholds. With ELISA 28 positives were identified but also a negative control returned a positive test result. It is known that endemic areas where past (high load) schistosome infections and poly-parasitism (e.g. with *Plasmodium* spp., and *Leishmania* spp. or other *helminthiases* [22]) are frequent, serological testing requires high specificity to avoid false-positive results. Whether the latter was due to the cross-reactive antibodies (frequently observed with other serological tests for schistosomiasis [23–25]), autoimmune antibodies [26–28], or an individual unaware of past exposure, could not be concluded. In fact antibody specificity may be difficult to confirm and verify when eggs or antigen detection are negative [29]. As an alleged better standard assay (in particular the HAMA-EITB, 100% specificity and sensitivity for *S. haematobium* detection; determined with confirmed cases [5] was not available it is difficult to conclude which of the two antibody tests applied in this pilot study is most accurate. However, we do believe that it is important to use at least two different serological antibody assays in parallel to achieve sufficient sensitivity in respect to the presence of *Schistosoma* antibody, demonstrating exposure. Assuming 100% sensitivity for the active infections in the serological antibody test protocol, all antibody positive samples would then require further testing with the UCP-LF CAA antigen assay to identify the active infections in this group.

The UCP-LF antigen assay detecting a *Schistosoma*-derived carbohydrate structure (CAA) in the human blood circulation (or other bodily fluids), identified two CAA-positive (clinically asymptomatic) individuals in the 37 previously cured schistosomiasis cases (infection registered between 1983 and 1994). Detection of CAA is an unequivocal proof for ongoing infection, the current presence of life worms as CAA regurgitated by the worms is rapidly (within hours) cleared from the human circulation, most likely via urine as the primary route [30]. In our study, determined CAA levels in sera were 4.2 and 3.5 pg/ml, a level indicative for low worm burden, potentially the presence of single worm-pair, or probably single sex (only male or only female) worms as no eggs are detected. There is some preliminary evidence indicating that in some cases (e.g. travelers) CAA serum levels below 1 pg/ml were observed (R. van Grootveld, 26^th^ECCMID 2016, Amsterdam, the Netherlands). In vitro studies with worms as well as studies with experimentally infected animals have indicated that a single worm pair would excrete a daily amount of CAA in the order of 40 ng, corresponding to 1–10 pg/ml blood [14]. Expectedly, both cases also tested antibody positive with ELISA confirming exposure/infection, but scored antibody negative with IHA. Besides above indicated issues with the IHA cutoff threshold, immunity to schistosome infections eventually leading to a decline in antibody response has been suggested in literature [31] and may explain the absence of antibody response when using IHA in these two cases as well. For one of the CAA-positive cases active infection was confirmed with a CAA-positive test result of the paired urine sample. The other CAA serum-positive was not confirmed with the applied urine test (UCAA950) and might have required a larger urine-volume for confirmation of CAA-positivity was not feasible due to the limited amount of sample that was made available for UCP-LF CAA testing at LUMC. As the paired urine sample was negative and not re-tested with a larger sample volume, one could challenge the positivity of this sample. However, a false positive signal is unlikely as the in serum detected level of CAA was well above the serum assay cutoff threshold but at a level that may not always result in a urine-positive test when testing with less than 2 mL of urine. Furthermore, PZQ treatment and follow-up testing would be the ultimate test to confirm disappearance of CAA positivity, this was not included in the protocol as participating individuals did not present any schistosomiasis clinical symptoms.

Clearly, positive UCP-LF CAA test results, whether obtained with serum or urine, do imply an ongoing infection although not necessarily involve egg shedding. Assuming no eggs were produced, the CAA-positive test result of the two egg-negative cases could the presence of: i) due to drug-treatment sterilized worm pairs; ii) single sex worms that survived past treatment; or less likely; iii) a low grade re-infection by single sex worms perhaps involving *S. bovis.* Factors as the presence of immature schistosome, high pre-treatment egg intensities (indicative of high worm burden), poor drug absorption and the short life time of PZQ, have been attributed to the reduced PZQ cure rate in some patients. Serum levels of circulating anodic antigen (CAA) can be directly related to actual worm burden worms. Low CAA concentrations indicate low worm burden and egg production can be easily missed with the common applied parasitological methods. As worms sometimes may recover testing after a somewhat longer period should be part of the protocol to verify the success of the drug treatment.

Schistosome worms do not multiply in the host, and in the absence of re-exposure the infection subsides when the schistosome worm dies, which is usually after 3–5 years [32]. However, the life span of a *S. haematobium* adult worm may be as long as 30 years [33]. Current study would support the latter potentially involving anti-fecundity immunity [34]. Alternatively Tata is a sympatric area of *S. bovis* and *S. haematobium*; phylogenetically, *S. haematobium*is ancestral to *S. bovis* and the ability to infect humans may have been retained by *S. bovis*. Because the human skin is thinner than the skin of a bovine, could be anticipated that *S. bovis* cercariae could penetrate a human without clinical signs. Moreover, the oviposition site of a potential schistosome hybrid pair is generally assumed to primarily be dependent on the species of the male worm and thus may end up in stool rather than urine [35, 36]. For the genus specific *Schistosoma* UCP-LF CAA test, the oviposition site doesn’t matter, urine is still an appropriate test matrix for detection of CAA regurgitated by the worm, independent of the pathway of egg clearance.

Applying the UCP-LF CAA test for *S. haematobium* diagnosis in a close-to-elimination setting in Zanzibar, United Republic of Tanzania, clearly confirmed that the empirical prevalence revealed with the UCP-LF CAA was several-fold higher that the prevalence detected with a single urine filtration [37]. Studies performed in People’s Republic of China confirmed the relevant higher prevalence when comparing egg counts (in stool) with the urine-antigen test for detection of *S. japonicum* infections [38]. Further studies must include molecular-species identification to understand a possible role of *S. bovis* infected snails in human *Schistosoma* infections *bovis-haematobium* sympatric areas where urogenital schistosomiasis is assume to be eliminated. Drug resistance and possible focal human *S. bovis* infection cannot be disregarded. Molecular-based approaches such as multiplex PCR based assaysnot only allow for distinction of different *Schistosoma* species as *S. bovis* and *S. haematobium* (and others species for immigrants/travelers) are useful for monitoring and optimizing control strategies and are also applicable for the simultaneous diagnosis of several other parasitic infections [39]. For larger (statistically more relevant) follow-up studies, stratified testing of high risk groups as e.g. fishermen should also be included. Evaluation of antigen positive tests using larger sample volume whenever possible should also be included. Moreover, testing of children born after transmission stop (2004) would provide important data regarding the efficiency of high sensitivity antibody testing in post-transmission settings.

## Conclusion

Morocco was successful in the elimination of urogenital schistosomiasis caused by *S. haematobium*. Since 2004, no new local cases were reported. However, the current study indicates that some individuals (last remaining cases) are still harboring *Schistosoma* worms which apparently are not shedding eggs; this demonstrates the need for a high sensitivity worm-antigen test as the UCP-LF CAA test. To prevent reemergence of schistosomiasis, national survey should focus on immigrants, travelers and all potential risk groups as e.g. children, professionally exposed individuals as canal cleaners, car washers and fishermen) directly with UCP-LF CAA independent of their antibody test result The UCP-LF CAA test identifies active infections of all *Schistosma* species, including veterinarian ones. In Morocco in sympatric *Schistosoma* areas as Tata, potential infection with *S. bovis* should be taken into consideration. The species identification in *Schistosoma* infected individuals (identified with the UCP-LF CAA test) would require a molecular approach.

## Additional file

Additional file 1: Multilingual abstracts in the six official working languages of the United Nations. (PDF 793 kb)

## Abbreviations

CAA: Circulating anodic antigen
IHA: Indirect haemagglutination assay
LF: Lateral flow
SCAA150: UCP-LF CAA test with 150 μl serum
TCA: Trichloroacetic acid
UCAA475: UCP-LF CAA test with 475 μl urine
UCP: Upconverting phosphor
