# Supplementary material for: Selecting accurate post-elimination monitoring tools to prevent reemergence of urogenital schistosomiasis in Morocco: a pilot study
Source: Infect Dis Poverty. 2017 Apr 6;6:75. doi: 10.1186/s40249-017-0289-z (PMC5382525; doi:10.1186/s40249-017-0289-z)
Supplement: Additional file 1: — Multilingual abstracts in the six official working languages of the United Nations. (PDF 793 kb) [file 40249_2017_289_MOESM1_ESM.pdf]

Translation of the abstract into the six official working languages of the United Nations

دراسة تجريبية : بعد القضاء على مرض البلهارسيا البولية التناسلية في المغرب: اختيار أدوات مراقبة دقيقة للتشخيص لمنع عودة ظهوره.

عبد العالي بالحبيب, فاطمة أمرير, ب ل أم كورستجن, س ج ددود, ج ج فاندام, أمينة حجلي, مريم بلحداد, بشرى منصوري, عبد الرحيم صادق, محمد الرجاوي, البشير عدلاوي.

ملخص:

بعد نجاح المملكة المغربية في القضاء على داء البلهارسيا البولية, التنزيل الفعلي لمرحلة ما بعد القضاء على المرض يجب استعمال اختبارات و تقنيات دقيقة لكشف الإصابات لأجل منع احتمال عودة الداء. واحة الرحالة الذي أجريت فيها هذه الدراسة سجلت آخر حالة مرض البلهارسيا البولية سنة 2004, و رغم هذا نجد أن % 30 من القواقع المائية لا تزال مصابة بالبلهارسيا البقرية, و هو ما يجعل احتمال إصابة الإنسان بها واردا. بالنسبة لتشخيص المرض بإجراء فحص عينة بولية أو براز للبحث على بيض البلهارسيا البولية فيبقى في المناطق الأقل انتشارا للداء أقل دقة, و بالتالي يبقى إجراء تحليل الدم للكشف عن مضادات الأجسام أو المستضدات الوسيلة الأنسب لرصد الداء.

للإجابة على هذا الإشكال قمنا بدراسة تجريبية و ذلك بمقارنة ثلاثة اختبارات: اختبارين متاحين تجاريا لرصد مضادات الأجسام ELISA و HAI و اختبار ثالث لرصد المستضدات UCP-LF CAA. أجربت هذه الاختبارات على عينات المصل و البول ل 37 مشاركا يقطنون بالرحالة بإقليم طاطا, هؤلاء المشاركين تم شفاؤهم بعدما أصيبوا بالمرض في الفترة ما بين 1982 و2003.

الاختبار البولي أكد عدم وجود أية بيضة للبلهارسيا عند جل المشاركين, بالنسبة لإختبارات مضادات الأجسام فقد بينت إختبارات HAI وجودها عند 6 مشاركين أما إختبار ELISA فبين وجودها بالنسبة ل 28 مع حالة واحدة مشكوك فيها . النتائج متطابقة في 18 حالة بين ELISA و HAI منها 5 من 6 حالات إيجابية باختبار HAI.

بالنسبة لإختبار الكشف عن المستضدات UCP-LF CAA التي أجريت علي المصل بينت وجود مستويات جد منخفضة (اقل 5pg/mL التي تدل على وجود دودة واحدة) بالنسبة لمشاركين شفيا قبل 21 و32 سنة وأما التي أجريت على البول فقد بينت وجود مستويات جد منخفضة في حالة واحدة من بين هذين المشاركين . الحالتين الايجابيتين باختبار UCP-LF CAA هما ايجابيتين أيضا بإختبار ELISA و سلبيتين بإختبار HAI .

من أجل منع عودة داء البلهارسيا البولية إلى المغرب فان برنامج الرصد المحلي يتطلب برتوكولات و اختبارات محددة و التي تشمل اختبار UCP-LF CAA الذي يسمح بتحديد حالات العدوى النشيطة كما يعتبر أداة تشخيصية لكشف درجة منخفضة لعدوى البلهارسيا بالنسبة للمسافربن و المهاجرين الذين تم شفاؤهم, الاختبار أيضا ممكن أن يحدد التهابات البلهارسيا البقرية.

كلمات أساسية

بلهارسيا, توقف العدوى, القضاء على الداء, التشخيص, اختبار الأجسام المضادة, اختبار المستضدات , CAA, عدوى نشيطة .

Translated from English version into Arabic by Abdelaali Balahbib, through

TRANSLATORS WITHOUT BORDERS

在摩洛哥选择使用精确的消除后监测工具来预防尿路血吸虫病的再度出现：一项试点研究

Abdelaali Balahbib, Fatima Amarir, Paul L.A.M. Corstjens, Claudia J. de Dood, Govert J.van Dam, Amina Hajli, Meryem Belhaddad, Bouchra El Mansouri, Abderrahim Sadak, Mohamed Rhajaoui and El Bachir Adlaoui

摘要

引言：在宣称要终止血吸虫病的传播和进一步降低消除后阶段的设定线，这需要有敏感的工具来监测血吸虫感染状况以便阻止血吸虫病的潜在复发。在 Rahala 地区自 2004 年就已经阻止了埃及血吸虫病的传播循环，但仍有 30%的钉螺被牛血吸虫（*Schistosoma bovis*）感染，潜在的人感染牛血吸虫并不能被排除。基于血吸虫虫卵数量检查的方法不能提供所需的灵敏度，设想将抗体或抗原筛查作为最合适的工具对此情况进行监测。

方法：在本试点研究中，比较 3 个检测方法的性能，即两个商业抗体检测（酶联免疫吸附试验和血细胞凝集方法）显示暴露情况，和 1 个抗原检测显示主动感染情况。所招募的 37 个参与者均居住在 Rahala（Akka, province Tata, Morocco），在 1983 年至 2003 年期间均被诊断患有血吸虫病且已治愈。在 2015 年这些无症状的参与者提供新鲜的临床样本（血液和尿液）用于上述检测。
结果：在 37 个参与者的尿液中均未检测到虫卵。血细胞凝集试验显示，阳性 8 例，然而 ELISA 检测有 28 人为阳性，1 个无法定性，1 个假阳性。ELISA 和血细胞凝集试验有 18 人匹配，包括 6 例血细胞凝集试验阳性中的 5 例。抗原测试（完成血样和尿样的比对）结果显示，两个参与者（已治愈 21 年和 32 年）的血清表明存在低水平的高特异性的循环阳极抗原，说明存在血吸虫感染水平低（小于 5 pg/m 与可能只有一对虫相符）。有 1 个尿样检测为循环阳极抗原阳性。ELISA 表明这两个 CAA 阳性病例存在人抗血吸虫抗体，血细胞凝集试验结果均为阴性。
结论：为了防止血吸虫病在摩洛哥再次出现，现有的监控程序需要特殊的步骤：包括对主动感染要进行 UCP-LPCAA 测试的抗体阳性检测，在旅行者、移民和已治愈的病例需采用合适的诊断工具来确定血吸虫的低度感染。属特异性检测也将用于鉴别与牛血吸虫相关的感染。

Translated from English version into Chinese by Yin-Long Li, edited by Pin Yang

CHINESE CENTER FOR DISEASE CONTROL AND PREVENTION
NATIONAL INSTITUTE OF PARASITIC DISEASES

Sélection d'outils de contrôle précis après l'élimination de la maladie pour empêcher la résurgence de la schistosomiase uro-génitale au Maroc: étude pilote

Abdelaali Balahbib, Fatima Amarir, Paul L.A.M. Corstjens, Claudia J. de Dood, Govert J.van Dam, Amina Hajli, Meryem Belhaddad, Bouchra El Mansouri, Abderrahim Sadak, Mohamed Rhajaoui et El Bachir Adlaoui

RÉSUMÉ

Contexte: une fois la transmission de la schistosomiase jugée terminée, puis après l'élimination de la maladie, des outils de dépistage sensibles sont nécessaires pour contrôler l'état de l'infection et empêcher une possible résurgence. A Rahala, même si le cycle de transmission de *Schistosoma haematobium* est interrompu depuis 2004, 30 % des escargots sont encore infectés par *S. bovis*. C'est pourquoi il ne faut pas exclure une possible infection humaine à *S. bovis*. Dans la mesure où les méthodes basées sur le dénombrement des œufs ne fournissent pas la sensibilité nécessaire, les analyses des anticorps ou des antigènes sont envisagées comme les outils les plus appropriés pour ce type de contrôle.
Méthodes: dans cette étude pilote, les performances de trois analyses ont été comparées. Deux tests de détection des anticorps disponibles dans le commerce (ELISA et l'hémagglutination) indiquant l'exposition, et un test de détection des antigènes (test chromatographique sur bandelette) démontrant une infection active. Les 37 participants à l'étude résidaient à Rahala (Akka, province de Tata, Maroc). Ils avaient été diagnostiqués et guéris d'une schistosomiase entre 1983 et 2003. En 2015, ces participants asymptomatiques ont fourni des échantillons cliniques frais (sang et urine) afin que des analyses soient réalisées à l'aide des tests de dépistage mentionnés ci-dessus.
Résultats: aucune présence d'œufs n'a été identifiée dans l'urine des 37 participants. Le test d'hémagglutination a indiqué 6 cas positifs de présence d'anticorps, alors que le test ELISA a indiqué 28 cas positifs de présence d'anticorps, ainsi qu'un cas incertain et un faux positif. Pour 18 participants, les résultats des tests ELISA et d'hémagglutination étaient identiques, y compris 5 cas sur les 6 positifs au test d'hémagglutination. Avec le test de détection des antigènes (effectué

sur des échantillons de sérum et d'urine appariés), le sérum de deux participants (guéris il y a 21 et 32 ans) indiquait la présence de faibles quantités de l'antigène anodique circulant de *schistosome* (CAA) qui est très spécifique. démontrant un faible taux d'infection parasitaire (moins de 5 pg / ml, soit probablement une seule paire de vers parasites). Un cas positif de CAA à également été détecté par le test d'urine chez un participant. Le test ELISA a révélé la présence d'anticorps *anti-schistosomes* humains chez ces deux cas positifs au CAA. Les tests d'hémagglutination étaient négatifs.

Conclusions: afin d'empêcher la résurgence de la schistosomiase au Maroc, les programmes de contrôle actuels nécessitent des protocoles spécifiques comme la détection des infections actives par l'analyse des résultats positifs à la présence d'anticorps avec le test de CAA UCP-LF, outil de diagnostic approprié pour identifier les faibles taux d'infection par *schistosome* chez les voyageurs, les immigrants et les personnes présumées guéries. Le test est spécifique au genre et permettra également d'identifier les infections liées au *S. bovis*.

Translated from English version into French by Emilie T., through

TRANSLATORS WITHOUT BORDERS

Выбор точных инструментов мониторинга в постликвидационный период для предотвращения повторного появления урогенитального шистосомоза в Марокко: пилотное исследование

Абделаали Балабиб, Фатима Амарир, Пол Л.А.М. Корстьенс, Клаудиа Дж. де Дуд, Говерт Дж. ван Дам, Амина Хаджли, Мерием Белхаддад, Бучра Эль-Мансури, Абдеррахим Садак, Мохамед Рхаджауи и Эль-Башир Адлауи

Аннотация

Базовая проблематика. После предполагаемого прекращения передачи шистосомоза и далее в ходе постликвидационного периода требуется использовать чувствительные инструменты для контроля состояния инфекции в целях предотвращения вероятности ее повторного появления. В Рахале, где цикл передачи *Schistosoma haematobium* прерван с 2004 года, но где 30% улиток по-прежнему заражены *S. bovis*, нельзя исключать появления потенциальной инфекции *S. bovis* у человека. Так как методы, основанные на подсчете количества яиц, не обеспечивают требуемой чувствительности, необходимо проводить анализы на наличие антител или антигенов, которые являются наиболее подходящими инструментами для этого вида мониторинга.

Методология. В данном пилотном исследовании были сопоставлены результаты трех проведенных анализов: двух присутствующих на рынке тестов на антитела (ELISA и реакции гемагглютинации), указывающих на наличие очага выделения инфекции, и теста на антигены (горизонтального проточного анализа), демонстрирующую наличие активной инфекции. Все 37 участников исследования проживали в Рахале (Акка, провинция Тата, Марокко). В период с 1983 по 2003 гг. у участников был диагностирован шистосомоз, от которого они получили соответствующее лечение. В 2015 году эти бессимптомные участники представили на анализ свежие клинические образцы (крови и мочи) для проведения вышеупомянутых диагностических тестов.

Результаты. В моче 37 участников не было выявлено ни одного яйца. Реакция гемагглютинации подтвердила наличие 6 серопозитивных лиц, в то время как тест ELISA выявил 28 серопозитивных лиц, кроме того, был получен один сомнительный и один ложноположительный результат. Результаты теста ELISA и реакции гемагглютинации совпали у 18 человек, среди которых 5 из 6 имеют положительную реакцию гемагглютинации.

В ходе проведению анализа антигенов (на основе парной сыворотки и образцов мочи), сыворотка двух участников (излеченных 21 и 32 года назад) указывала на наличие низких уровней циркулирующего анодного антигена (CAA) *Schistosoma,* что свидетельствует о присутствии инфекции с малой концентрацией паразитов в организме (менее 5пг/мл, что, вероятно, соответствует одной паре червей). У одного из участников положительный CAA был зафиксирован в моче. В обоих случаях с положительным CAA результаты теста ELISA показали присутствие антител к человеческой разновидности*Schistosoma,* при этом результаты реакции гемагглютинации были отрицательными.

Выводы. Для того чтобы предотвратить повторное появление шистосомоза в Марокко, в рамках существующих программ мониторинга необходимо разработать специальные протоколы, которые включают в себя проверку серопозитивных участников на наличие активной инфекции с помощью теста CAA UCP-LF — соответствующего диагностического инструмента для выявления случаев заражения *Schistosoma* среди путешественников, иммигрантов и лиц, ранее прошедших лечение, с низким уровнем концентрации инфекции. Тест предназначен для определения родовой принадлежности вируса и также выявляет инфекции, связанные с *S. bovis*.

Translated from English version into Russian by Irina Zayonchkovskaya, through

TRANSLATORS WITHOUT BORDERS

Selección de herramientas precisas de control posterior a la eliminación para prevenir la reaparición de la esquistosomiasis urogenital en Marruecos: estudiopiloto

Abdelaali Balahbib, Fatima Amarir, Paul LAM Corstjens, Claudia J. de Dood, Govert J. van Dam, Amina Hajli, Meryem Belhaddad, Bouchra El Mansouri, Abderrahim Sadak, Mohamed Rhajaoui y El Bachir Adlaoui

RESUMEN

Antecedentes: Tras la presunta detención de la transmisión de la esquistosomiasis y más adelante en el ámbito posterior a la eliminación, hacen falta herramientas sensibles para vigilar la situación de la infección y prevenir su posible reaparición. En Rahala, donde el ciclo de transmisión del *Schistosoma haematobium* está interrumpido desde 2004, pero donde el 30% de los caracoles todavía están infectados por *S. bovis*, no se puede excluir una posible infección por *S. bovis* en humanos. Como los métodos basados en el recuento de huevos no proporcionan la sensibilidad que es precisa, se prevé que los análisis para la detección de anticuerpos o antígenos sean las herramientas más adecuadas para este tipo de vigilancia.

Métodos: En este estudio piloto, se comparó la eficacia de tres análisis: dos pruebas de detección de anticuerpos (ELISA y formato de hemaglutinación) disponibles comercialmente que indican la exposición, y una prueba de detección de antígenos (formato de tira de flujo lateral) que demuestra una infección activa. Los 37 participantes que se incluyeron en el estudio residían en Rahala (Akka, provincia de Tata, Marruecos). A los participantes se les había diagnosticado y se habían curado de esquistosomiasis en el período entre 1983 y 2003. En 2015 estos participantes asintomáticos proporcionaron nuevas muestras clínicas (de sangre y orina) para su análisis con las pruebas de diagnóstico antes mencionados.

Resultados: No se identificaron huevos en la orina de los 37 participantes. La prueba de hemaglutinación arrojó 6 resultados positivos para anticuerpos, mientras que el ELISA indicó 28 resultados positivos, uno no concluyente y un falso positivo. Los resultados de ELISA y hemaglutinación coincidieron para 18 personas, entre las cuales 5 de 6 dieron positivo por hemaglutinación. Con la prueba para la detección de antígenos (realizada en muestras pareadas de suero y orina), el suero de dos participantes (curados hace 21 y 32 años) indicó la presencia de niveles bajos del antígeno circulante anódico (CAA) muy específico *Schistosoma*, lo que demuestra infecciones por bajos niveles de gusanos (menos de 5 pg / ml correspondientes a probablemente un solo par de gusanos). El resultado de uno dio también positivo para el CAA en la orina. La prueba ELISA indicó la presencia de anticuerpos anti-*Schistosoma* humanos en estos dos casos positivos para CAA, siendo negativos los resultados por hemaglutinación.

Conclusiones: Para prevenir la reaparición de la esquistosomiasis en Marruecos, los programas de control actuales requieren protocolos específicos que incluyan el análisis de los casos positivos para anticuerpos con el fin de detectar una infección activa mediante la prueba de CAA UCP-LF, la herramienta de diagnóstico adecuada para identificar infecciones por *Schistosoma* de bajo nivel en viajeros, inmigrantes y presuntos casos curados. La prueba, que es específica de género, también identificará las infecciones por *S. bovis*.

Translated from English version into Spanish by Alicia Vaquero, through

TRANSLATORS
WITHOUT BORDERS
